# Association Between Physical Activity Levels and Life Functioning in Korean Older Adults: A Cross-Sectional Analysis of the 2024 Korea National Health and Nutrition Examination Survey

**DOI:** 10.3390/healthcare14182965

**Published:** 2026-09-11

**Authors:** Namkuk Son

**Affiliations:** Department of Sports Welfare, Kyungil University, Gyeongsan 38428, Republic of Korea; 1103209@kiu.kr; Tel.: +82-53-600-5915

**Keywords:** KNHANES, life functioning, muscle-strengthening exercise, older adults, physical activity

## Abstract

**Highlights:**

**What are the main findings?**

- Meeting the recommended levels of aerobic physical activity, muscle-strengthening exercise, or both was associated with better overall life functioning and lower-extremity function.
- Associations with upper-extremity function, instrumental activities of daily living, and social participation varied across physical activity groups and outcome specifications.

**What are the implications of the main findings?**

- Meeting aerobic physical activity and muscle-strengthening exercise recommendations was associated with better life-functioning profiles in older adults.
- These associations should be interpreted cautiously because the cross-sectional design does not establish the direction or causality of the observed relationships.

**Abstract:**

**Background/Objectives**: The purpose of this study was to investigate the association between physical activity levels and life functioning among Korean older adults using data from the 2024 Korea National Health and Nutrition Examination Survey (KNHANES). **Methods**: Data from 1418 adults aged ≥65 years were analyzed in this cross-sectional study. Participants were classified into Aerobic-only, Muscle-strengthening-only, Combined, and Inactive groups according to whether their self-reported physical activity met the World Health Organization’s recommendations. Associations with life functioning were examined using complex sample logistic regression analyses adjusting for demographic, lifestyle, and health-related covariates. **Results**: Compared with the Inactive group, the Aerobic-only (odds ratio [OR] 1.55, 95% confidence interval [CI] 1.11–2.18), Muscle-strengthening-only (OR 1.65, 95% CI 1.06–2.56), and Combined groups (OR 2.19, 95% CI 1.41–3.39) had significantly higher odds of having no limitations in overall life functioning. Similar associations were observed for lower-extremity function, while associations with other functional domains varied across physical activity groups. **Conclusions**: In this cross-sectional study, meeting the recommended levels of aerobic physical activity, muscle-strengthening exercise, or both was associated with higher odds of having no limitations in overall life functioning and lower-extremity function.

## 1. Introduction

Population aging has accelerated worldwide, primarily due to declining fertility rates and increasing life expectancy [1,2,3]. In the Republic of Korea, adults aged 65 years and older accounted for 20.3% of the total population in 2025, and this proportion is projected to continue increasing [4]. However, increases in life expectancy do not necessarily translate into increases in healthy life expectancy, suggesting that individuals may spend a longer period in later life living with disease or disability [5]. In 2024, life expectancy in Korea was 83.7 years, whereas healthy life expectancy was 65.5 years, indicating that Koreans were estimated to spend an average of 18.2 years living with disease or disability [6]. Because a longer duration of living with disease is associated with a greater burden of comorbidities, reduced quality of life, and increased healthcare costs, efforts to extend healthy life expectancy are needed [7,8]. Aging is generally accompanied by a decline in physical activity, which may contribute to functional limitations, loss of independence, and an increased risk of chronic disease [2,9]. In particular, age-related loss of muscle mass contributes to declines in muscle strength and physical function [10,11].

Physical function refers to the ability to perform physical activities required for daily living and encompasses motor function and control, physical fitness, and mobility, which refers to the ability to move independently from one place to another [12,13]. Therefore, even among individuals of the same chronological age, levels of physical function may vary, which can be described in terms of functional age [3]. Because physical function is closely associated with quality of life, appropriate assessment of physical function in older adults is essential for healthy aging [3,14]. Given that declines in physical function are generally known to begin in midlife, around the fourth or fifth decade of life, early identification of such declines and the implementation of appropriate interventions may play an important role in preventing further deterioration in physical function in later life and improving overall quality of life [3,15].

The World Health Organization defines physical activity as any bodily movement produced by skeletal muscles that requires energy expenditure, whereas physical inactivity refers to an insufficient level of physical activity below the recommended levels [16]. A decline in physical activity may lead to reduced physical function, while reduced physical function may further limit participation in physical activity, suggesting a bidirectional relationship between physical activity and physical function [15]. Therefore, increasing regular physical activity may contribute to improvements in physical function, which may in turn facilitate greater participation in physical activity. Several previous studies in older adults have reported associations between higher levels of physical activity and better physical function [17,18,19].

However, most previous studies have assessed physical function using performance-based measures that require participants to directly perform specific tasks. Widely used assessment tools include the Functional Independence Measure, Short Physical Performance Battery (SPPB), and Senior Fitness Test [3,20]. These tools can comprehensively assess various aspects of physical function, including the ability to perform activities of daily living, mobility, balance, and muscle strength. In addition, individual measures such as handgrip strength, the chair stand test, and gait speed are widely used to assess specific aspects of physical function [3,20]. Although these performance-based assessments have the advantage of providing relatively objective and accurate measurements through direct assessment of participants, their application to large population-based studies may be limited because they require a considerable amount of time and resources.

Interviewer-administered or self-administered assessment tools include the Barthel Index, Katz Index, and 36-Item Short Form Health Survey (SF-36) Physical Functioning Subscale [21]. In Korea, the Life Functioning (LF) scale was developed to assess functional ability among adults aged 50 years and older by incorporating physical function, activities of daily living, and social participation [22]. Unlike the PF scale, which evaluates functional limitations and disability without assessing social activities, the LF scale incorporates social activities, thereby providing a broader assessment of functional ability [22]. As a self-administered questionnaire, the LF scale has the advantage of enabling the assessment of overall life functioning in a large number of individuals within a relatively short period of time. Although the scale was originally developed with 25 items, a shortened 10-item version (LF-10) was used in the 2024 Korea National Health and Nutrition Examination Survey (KNHANES).

Because the LF-10 was first introduced in the 2024 KNHANES, evidence regarding life functioning assessed using the LF-10 among older adults remains limited. The LF-10 assesses life functioning across multiple domains, including physical function, instrumental activities of daily living, and social participation. The WHO physical activity guidelines provide separate recommendations for aerobic physical activity and muscle-strengthening exercise [16]. Recent population-based research in older adults has similarly evaluated adherence to aerobic and muscle-strengthening recommendations separately and in combination [23]. Thus, distinguishing older adults based on their adherence to these recommendations allows the associations of aerobic physical activity and muscle-strengthening exercise, separately and in combination, with life functioning to be examined. However, it remains unclear whether meeting these recommendations is associated with life functioning assessed using the LF-10 among Korean older adults.

Accordingly, this study aimed to investigate the association between physical activity levels and life functioning among Korean older adults using data from the 2024 KNHANES. It was hypothesized that meeting these recommendations would be positively associated with lower- and upper-extremity function, instrumental activities of daily living (IADL), and social participation, with the strongest associations among older adults meeting both recommendations.

## 2. Materials and Methods

### 2.1. Data Source and Participants

**Figure 1:**
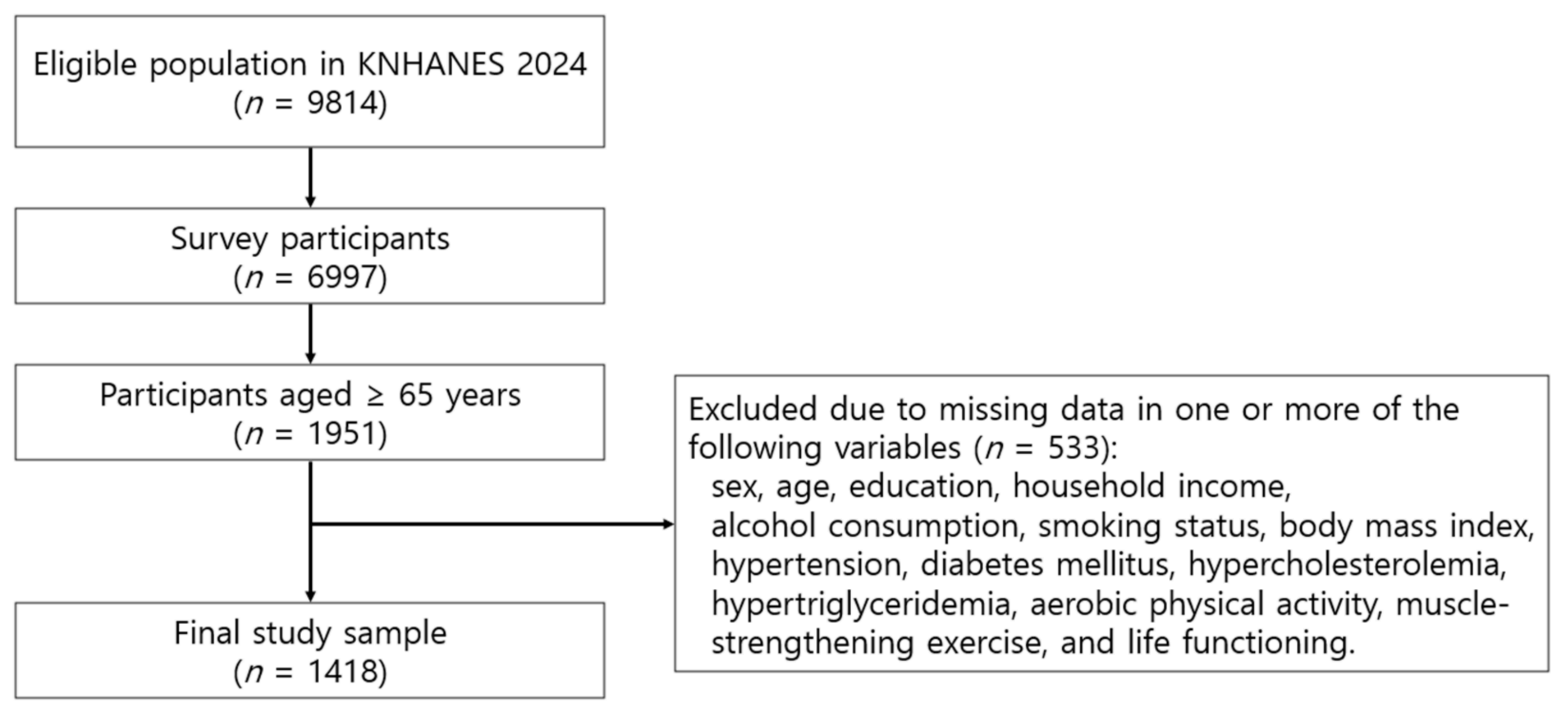
Flowchart of participant selection from the 2024 Korea National Health and Nutrition Examination Survey (KNHANES).

This study analyzed raw data from the 2024 Korea National Health and Nutrition Examination Survey (KNHANES), a nationally representative survey conducted by the Korea Disease Control and Prevention Agency (KDCA) [24]. The 2024 KNHANES was approved by the Institutional Review Board of the KDCA (No. 2022-11-16-R-03) [25]. Of the 9814 individuals selected for the survey, 6997 participated in at least one of the health interview, health examination, or nutrition surveys, of whom 1951 were aged 65 years or older. The variables included in the present analysis were sex, age, education level, household income, alcohol consumption, smoking status, and body mass index as general characteristics; the presence of hypertension, diabetes mellitus, hypercholesterolemia, and hypertriglyceridemia as chronic disease-related variables; and adherence to the recommended level of aerobic physical activity, number of days of muscle-strengthening exercise, and the LF-10 as physical activity and life functioning-related variables. After excluding 533 participants with missing data for any of these variables, 1418 participants were included in the final analysis (Figure 1). As this study involved a secondary analysis of existing data, it was granted an exemption from review by the Institutional Review Board of Kyungil University (No. 1041459-202607-HR-014-01).

### 2.2. Study Variables

#### 2.2.1. General Characteristics

The general characteristics of the participants included sex, age, education level, household income, alcohol consumption, smoking status, and body mass index (BMI) (Table 1). All variables were categorized for analysis. Age was categorized as 65–69, 70–74, 75–79, and ≥80 years. Education level was categorized as elementary school or lower, middle school, high school, and college or higher. Household income was categorized into quartiles (high, upper-middle, lower-middle, and low). Alcohol consumption was categorized as less than once per month (including lifetime abstainers) or at least once per month during the past year. Smoking status was categorized as current non-smokers or current smokers based on the current use of conventional cigarettes, heated tobacco products, or e-cigarettes. BMI was categorized as underweight (<18.5 kg/m^2^), normal weight (≥18.5 to <25 kg/m^2^), or obesity (≥25 kg/m^2^). Hypertension, diabetes mellitus, hypercholesterolemia, and hypertriglyceridemia were included as chronic disease-related variables and categorized as present or absent (Table 1).

#### 2.2.2. Life Functioning

The LF-10 consists of 10 items, each rated using five response categories scored from 0 to 4, yielding a maximum total raw score of 40 points. However, because the KNHANES converts the total LF-10 score to a 100-point scale, the converted score was used in this study. Participants were classified into two groups: those with a score of 100, indicating no limitations in overall life functioning, and those with a score below 100, indicating limitations in overall life functioning. The LF-10 items were further categorized into four domains: lower-extremity function (5 items; maximum score, 20 points), upper-extremity function (2 items; maximum score, 8 points), instrumental activities of daily living (IADL; 2 items; maximum score, 8 points), and social participation (1 item; maximum score, 4 points). For each domain, participants were classified into those with the maximum score, indicating no limitation, and those with a score below the maximum, indicating a limitation. Detailed information on the individual LF-10 items is presented in Table 2.

All items were preceded by the following question: “How much difficulty do you have carrying out a particular activity without the help of someone else or the use of assistive devices?”. Responses were rated on a 5-point Likert scale: 0 = cannot do, 1 = a lot, 2 = some, 3 = a little, and 4 = none.

#### 2.2.3. Physical Activity Levels

Aerobic physical activity was classified according to whether participants met the WHO recommendation of at least 150 min of moderate-intensity physical activity per week, at least 75 min of vigorous-intensity physical activity per week, or an equivalent combination of moderate- and vigorous-intensity physical activity [16]. Muscle-strengthening exercise was classified according to whether participants performed muscle-strengthening exercise on at least 2 days per week, in accordance with the WHO recommendation, or on fewer than 2 days per week [16]. Based on these criteria, participants were categorized into four physical activity groups: the Combined group, which met both the aerobic physical activity and muscle-strengthening exercise recommendations; the Aerobic-only group, which met only the aerobic physical activity recommendation; the Muscle-strengthening-only group, which met only the muscle-strengthening exercise recommendation; and the Inactive group, which met neither recommendation.

#### 2.2.4. Covariates

The covariates included sex, age, education level, household income, alcohol consumption, smoking status, body mass index (BMI), hypertension, diabetes mellitus, hypercholesterolemia, and hypertriglyceridemia. Covariates were selected a priori to account for demographic, socioeconomic, lifestyle-related, and health-related characteristics that could be related to physical activity and life functioning in older adults. Sex and age were included as demographic factors, education level and household income as socioeconomic factors, and alcohol consumption, smoking status, and BMI as lifestyle- and health-related factors. Hypertension, diabetes mellitus, hypercholesterolemia, and hypertriglyceridemia were additionally included to account for differences in chronic health status.

### 2.3. Statistical Analysis

The KNHANES employs a two-stage stratified cluster sampling design. Therefore, all analyses were conducted using complex sample procedures that accounted for strata, clusters, and sampling weights. Specifically, kstrata was used as the stratification variable, PSU as the cluster variable, and wt_itvex as the sampling weight. Adults aged ≥65 years with complete data for the study variables were analyzed as a subpopulation within the complex survey design rather than being filtered from the dataset before variance estimation.

For the four physical activity groups, unweighted frequencies and weighted percentages were calculated for sex, age, education level, household income, alcohol consumption, smoking status, body mass index, prevalence of hypertension, diabetes mellitus, hypercholesterolemia, and hypertriglyceridemia, and LF-10 score levels. Complex sample frequency analysis and the Rao–Scott χ^2^ test were used to examine significant differences in proportions across the physical activity groups while accounting for the complex sampling design. To examine the associations between physical activity levels and life functioning in older adults, complex sample logistic regression analyses were performed. The Inactive group, which met neither the recommended level of aerobic physical activity nor that of muscle-strengthening exercise, was used as the reference group. Odds ratios (ORs) and 95% confidence intervals (CIs) were calculated for having “no limitation” in each life functioning domain for each physical activity group. In addition, as a sensitivity analysis, complex sample general linear models were used to analyze the LF-10 total and domain scores without dichotomization. The Inactive group was used as the reference group, and unstandardized regression coefficients (B) with 95% CIs were estimated for each physical activity group using the same covariate adjustment as in the fully adjusted logistic regression model. All statistical analyses were performed using SPSS version 32.0 (IBM Corp., Armonk, NY, USA), and the level of statistical significance was set at 0.05.

## 3. Results

### 3.1. General Characteristics by Physical Activity Group

The unweighted frequencies and weighted percentages of variables related to general characteristics according to physical activity group are presented in Table 1. Significant differences among the groups were observed for sex, age, education level, household income level, alcohol consumption, and prevalence of hypertriglyceridemia. The proportion of men was highest in the Combined group (70.6%), whereas it was lower than 40% in the Inactive and Aerobic-only groups. Regarding age distribution, the proportion of participants aged 65–69 years was highest in the Combined and Aerobic-only groups (44.6%), whereas the proportion of those aged ≥80 years was highest in the Inactive group (21.5%). Regarding education level, the proportion of participants with an elementary school education or lower was lowest in the Combined group (21.2%) and highest in the Inactive group (48.5%). In contrast, the proportion of participants with a college education or higher was highest in the Combined group (26.3%) and lowest in the Inactive group (8.9%). Regarding household income level, the proportion of participants in the highest income category was highest in the Combined group (16.7%) and lowest in the Inactive group (10.4%). Conversely, the proportion in the lowest income category was lowest in the Combined group (18.8%) and highest in the Inactive group (38.7%). The proportion of participants who consumed alcohol at least once per month was highest in the Combined group (51.1%) and lowest in the Inactive group (29.9%). No significant differences among the groups were observed in smoking status or body mass index. Regarding chronic diseases, the prevalence of hypertriglyceridemia was lowest in the Muscle-strengthening-only group (3.9%) and highest in the Inactive group (10.3%). However, no significant differences among the groups were observed in the prevalence of hypertension, diabetes mellitus, or hypercholesterolemia.

### 3.2. Life Functioning Levels by Physical Activity Group

The unweighted frequencies and weighted percentages for each LF-10 domain according to physical activity group are presented in Table 3. Significant differences among the groups were observed for the LF-10 total score and all individual domains. The proportion of participants achieving the maximum LF-10 total score was highest in the Combined group (54.0%) and lowest in the Inactive group (23.9%). Similar patterns were observed across all individual domains. The proportions of participants achieving the maximum scores for lower-extremity function, upper-extremity function, IADL, and social participation were highest in the Combined group, at 55.7%, 88.5%, 96.5%, and 94.6%, respectively, and lowest in the Inactive group, at 25.5%, 65.3%, 77.7%, and 82.4%, respectively.

### 3.3. Association Between Physical Activity and Life Functioning

The results of the logistic regression analyses examining the association between physical activity levels and life functioning are presented in Table 4. After adjustment for covariates (Model 3), significantly higher odds of having no limitation in overall life functioning were observed in the Aerobic-only group (OR: 1.55, 95% CI: 1.11–2.18), Muscle-strengthening-only group (OR: 1.65, 95% CI: 1.06–2.56), and Combined group (OR: 2.19, 95% CI: 1.41–3.39) compared with the Inactive group. All three physically active groups also had significantly higher odds of having no limitation in lower-extremity function. Significant associations with upper-extremity function, IADL, and social participation were observed for some physical activity groups, as detailed in Table 4. The confidence intervals for some estimates, particularly those for IADL, were wide, indicating limited precision.

### 3.4. Sensitivity Analysis of the Associations Between Physical Activity and LF-10 Scores

The results of the sensitivity analyses using LF-10 total and domain scores without dichotomization are presented in Table 5. In the fully adjusted models, significantly higher LF-10 total scores were observed in the Aerobic-only (B = 3.31, 95% CI: 1.48–5.14), Muscle-strengthening-only (B = 5.55, 95% CI: 3.20–7.90), and Combined groups (B = 4.26, 95% CI: 2.31–6.21) compared with the Inactive group. All three physically active groups also had significantly higher lower-extremity function and IADL scores. For upper-extremity function, significantly higher scores were observed in the Muscle-strengthening-only and Combined groups, whereas for social participation, significantly higher scores were observed in the Aerobic-only and Muscle-strengthening-only groups. Compared with the primary logistic regression analyses, the sensitivity analyses additionally showed significant associations of the Muscle-strengthening-only group with upper-extremity function and social participation, and of the Aerobic-only group with IADL. Overall, the sensitivity analyses showed generally consistent associations with the primary analyses, although some domain-specific patterns differed.

## 4. Discussion

This study examined the association between physical activity levels and life functioning among adults aged ≥65 years using data from the 2024 KNHANES. The LF-10, which was used to assess life functioning in this study, is an instrument designed to evaluate functional limitations in the upper and lower extremities, as well as disabilities in activities of daily living and social participation [22]. The results of Model 3, adjusted for covariates, can be summarized as follows. Compared with the Inactive group, all three physically active groups had significantly higher odds of having no limitation in overall life functioning and lower-extremity function. In addition, the Combined group had significantly higher odds of having no limitation in upper-extremity function, while the Muscle-strengthening-only and Combined groups had significantly higher odds of having no limitation in IADL. For social participation, significantly higher odds of having no limitation were observed in the Aerobic-only group compared with the Inactive group. Sensitivity analyses using the LF-10 total and domain scores without dichotomization generally supported the primary findings, although some domain-specific patterns differed. Specifically, additional significant associations were observed for the Muscle-strengthening-only group with upper-extremity function and social participation and for the Aerobic-only group with IADL. These findings were generally consistent with our hypothesis that meeting aerobic physical activity and muscle-strengthening exercise recommendations would be positively associated with life functioning, with the Combined group showing the highest odds ratio for overall life functioning. However, the differences in domain-specific patterns between the primary and sensitivity analyses indicate that associations for individual domains should be interpreted cautiously. Overall, the observed associations suggest that meeting recommendations for aerobic physical activity, muscle-strengthening exercise, or both was associated with fewer limitations in life functioning among Korean older adults. However, given the cross-sectional design, these associations may also reflect the possibility that older adults with better life functioning were more able to meet the physical activity recommendations, rather than physical activity leading to better life functioning.

Aerobic physical activity includes activities such as brisk walking, running, cycling, and swimming that increase heart rate and breathing and generally involve sustained movement of large muscle groups [16,26]. Previous studies have reported that aerobic physical activity is associated with improvements in lower-extremity strength, balance, walking ability, and performance-based measures of physical function, including the Timed Up and Go test and the SPPB [27,28,29,30]. These findings are consistent with the significant association between the Aerobic-only group and lower-extremity function observed in the present study. Resistance exercise has also been reported to improve muscle strength and neuromuscular function in older adults [31,32,33], which is consistent with the association observed for the Muscle-strengthening-only group. More recently, a systematic review and network meta-analysis of 151 randomized trials reported beneficial effects of resistance training on physical function and lower-extremity muscle strength in older adults [34]. However, an 8-month adapted taekwondo program incorporating strength and balance exercises also demonstrated differential changes in functional fitness, with improvements in aerobic endurance and coordination, whereas other physical fitness measures showed no significant changes [35]. In the present study, the Combined group also had significantly higher odds of having no limitation in lower-extremity function than the Inactive group.

To maintain functional independence, older adults need to adequately perform activities of daily living involving the upper extremities, such as eating, washing, and cleaning. A systematic review reported that resistance exercise performed for at least 4 weeks improved postural tremor, force steadiness, and manual dexterity in healthy older adults [31]. The upper-extremity function domain of the LF-10 includes activities requiring the manipulation of small objects and lifting a 5 kg object. In the present study, the Combined group had significantly higher odds of having no limitation in upper-extremity function compared with the Inactive group. In the sensitivity analysis using the upper-extremity function score without dichotomization, significantly higher scores were also observed in the Muscle-strengthening-only group compared with the Inactive group. Taken together, these findings suggest that meeting the muscle-strengthening exercise recommendation, either alone or in combination with the aerobic physical activity recommendation, may be associated with better upper-extremity functioning.

The IADL domain of the LF-10 consists of two items, bathing or showering and using public transportation, both of which require multiple aspects of physical function. Previous studies reporting improvements in muscle and neuromuscular function following resistance exercise provide relevant context for these observed associations [31,32,33]. In the present study, the Muscle-strengthening-only and Combined groups had significantly higher odds of having no limitation compared with the Inactive group. The sensitivity analysis additionally showed a significantly higher IADL score in the Aerobic-only group compared with the Inactive group. Taken together, the primary and sensitivity analyses suggest that better IADL functioning was associated with all three physically active groups compared with the Inactive group.

In a study of 1146 older adults with a mean age of 70.1 years, Kikuchi et al. [36] reported that higher levels of social participation were associated with higher levels of moderate-to-vigorous physical activity in both men and women. In the present study, the Aerobic-only group had significantly higher odds of having no limitation in social participation compared with the Inactive group. In the sensitivity analysis using the social participation score without dichotomization, a significantly higher score was also observed in the Muscle-strengthening-only group compared with the Inactive group. Taken together, the primary and sensitivity analyses suggest that better social participation was associated with the Aerobic-only and Muscle-strengthening-only groups compared with the Inactive group. However, these findings should be interpreted cautiously because social participation was assessed using a single LF-10 item, and the 95% confidence interval for the Aerobic-only group in the primary analysis was 1.02–2.29 (OR: 1.53).

This study has several limitations. First, because of the cross-sectional design, causal relationships between physical activity levels and life functioning cannot be established. That is, although physical activity may contribute to the maintenance of life functioning, the possibility of reverse causation, whereby older adults with better life functioning are more likely to engage in higher levels of physical activity, cannot be excluded. Second, physical activity levels were assessed using self-reported questionnaires and may therefore have been subject to recall bias and measurement error. In addition, information on the specific types and intensity of physical activity, the settings in which the activities were performed, and whether they involved interaction with others was unavailable, limiting a more detailed interpretation of the associations with different types of physical activity. Third, the LF-10 total and domain scores were dichotomized according to whether the maximum score was achieved. This approach may have resulted in loss of information and limited the ability to detect differences in the degree of functioning among older adults. To address this limitation, sensitivity analyses were conducted using the LF-10 total and domain scores without dichotomization. These analyses generally supported the primary findings, although some domain-specific patterns differed, indicating that associations for individual domains should be interpreted cautiously. Furthermore, social participation was assessed using a single item, which may not have adequately captured the diverse characteristics of this domain. Fourth, 533 eligible older adults were excluded from the analysis because of missing data. This exclusion may have introduced selection bias if the excluded participants differed systematically from those included in the analysis. In addition, the small number of participants with limitations in some subgroup and domain combinations resulted in wide confidence intervals, indicating limited precision of some estimates. Fifth, because several “no limitation” outcomes were highly prevalent, the ORs may overstate the magnitude of the corresponding prevalence associations. Therefore, the ORs should be interpreted as measures of association rather than as approximations of prevalence ratios. Future studies should therefore use objectively measured physical activity and longitudinal data and consider the specific types and settings of physical activity as well as detailed characteristics of social participation.

## 5. Conclusions

In this cross-sectional study of Korean older adults, meeting the recommended level of aerobic physical activity, muscle-strengthening exercise, or both was associated with higher odds of having no limitation in overall life functioning and lower-extremity function. In addition, meeting both recommendations was associated with higher odds of having no limitation in upper-extremity function and IADL. Overall, adherence to these physical activity recommendations was associated with better life-functioning profiles among Korean older adults. Further longitudinal and intervention studies are needed to clarify the temporal and causal relationships between physical activity and life functioning.

## Figures and Tables

**Table 1:** General characteristics of participants by physical activity group.

		Combined *n* = 151 (11.7)	Muscle-Strengthening Only *n* = 204 (14.5)	Aerobic Only *n* = 300 (22.1)	Inactive *n* = 763 (51.7)	*p*
Sex	Male	103 (70.6)	116 (58.1)	104 (36.7)	275 (38.7)	<0.001
	Female	48 (29.4)	88 (41.9)	196 (63.3)	488 (61.3)	
Age	65–69	60 (44.6)	57 (27.6)	121 (44.6)	233 (33.2)	<0.001
	70–74	51 (31.5)	61 (25.4)	90 (25.6)	217 (24.2)	
	75–79	30 (17.5)	56 (29.4)	49 (16.6)	155 (21.1)	
	80 or older	10 (6.3)	30 (17.6)	40 (13.2)	158 (21.5)	
Educational status	Elementary school or lower	30 (21.2)	74 (35.8)	128 (41.8)	384 (48.5)	<0.001
	Middle school	26 (17.1)	33 (14.6)	67 (23.0)	172 (23.0)	
	High school	54 (35.5)	58 (27.8)	73 (23.9)	144 (19.6)	
	College or higher	41 (26.3)	39 (21.8)	32 (11.3)	63 (8.9)	
Household income (quartile)	Low	30 (18.8)	74 (34.3)	115 (35.2)	316 (38.7)	0.006
	Mid-low	55 (36.8)	61 (31.2)	104 (36.1)	231 (30.9)	
	Mid-high	41 (27.7)	44 (22.6)	46 (16.0)	148 (20.0)	
	High	25 (16.7)	25 (11.9)	35 (12.7)	68 (10.4)	
Alcohol drinking	Yes	74 (51.1)	77 (37.7)	106 (36.8)	224 (29.9)	<0.001
	No	77 (48.9)	127 (62.3)	194 (63.2)	539 (70.1)	
Smoking	Yes	9 (5.7)	13 (5.2)	22 (9.0)	66 (8.3)	0.400
	No	142 (94.3)	191 (94.8)	278 (91.0)	697 (91.7)	
Body mass index	Underweight	6 (4.2)	7 (4.1)	8 (2.8)	26 (3.2)	0.393
	Normal	103 (64.6)	115 (59.1)	189 (65.2)	443 (58.2)	
	Obese	42 (31.3)	82 (36.8)	103 (32.0)	294 (38.6)	
Hypertension	Yes	84 (54.1)	130 (64.1)	170 (55.8)	492 (63.7)	0.076
	No	67 (45.9)	74 (35.9)	130 (44.2)	271 (36.3)	
Diabetes mellitus	Yes	41 (30.2)	57 (27.3)	87 (30.3)	238 (31.4)	0.796
	No	110 (69.8)	147 (72.7)	213 (69.7)	525 (68.6)	
Hypercholesterolemia	Yes	72 (46.7)	95 (47.0)	151 (50.9)	363 (48.2)	0.795
	No	79 (53.3)	109 (53.0)	149 (49.1)	400 (51.8)	
Hypertriglyceridemia	Yes	7 (5.1)	9 (3.9)	18 (6.0)	77 (10.3)	0.004
	No	144 (94.9)	195 (96.1)	282 (94.0)	686 (89.7)	

Values are presented as unweighted frequencies (*n*) and weighted percentages (%). *p*-values were obtained using the Rao–Scott chi-square test.

**Table 2:** Items and response categories of the Life Functioning scale-10 (LF-10).

Domain	Item
Lower extremity function	1. Standing up from a chair
	2. Standing on tiptoes
	3. Bending, squatting, or kneeling
	4. Walking 400 m
	5. Climbing one floor
Upper extremity function	6. Grasping small objects
	7. Lifting 5 kg
Instrumental activities of daily living (IADL)	8. Bathing or showering
	9. Using public transportation
Social participation	10. Participating in social activities

**Table 3:** Life functioning levels of participants by physical activity group.

		Combined *n* = 151 (11.7)	Muscle-Strengthening Only *n* = 204 (14.5)	Aerobic Only *n* = 300 (22.1)	Inactive *n* = 763 (51.7)	*p*
Total score	<100	70 (46.0)	132 (62.2)	204 (65.5)	585 (76.1)	<0.001
	100	81 (54.0)	72 (37.8)	96 (34.5)	178 (23.9)	
Lower extremity function score	<20	67 (44.3)	128 (60.4)	199 (63.6)	571 (74.5)	<0.001
	20	84 (55.7)	76 (39.6)	101 (36.4)	192 (25.5)	
Upper extremity function score	<8	21 (11.5)	49 (22.6)	105 (32.4)	275 (34.7)	<0.001
	8	130 (88.5)	155 (77.4)	195 (67.6)	488 (65.3)	
Instrumental activities of daily living score	<8	7 (3.5)	20 (9.0)	48 (15.3)	173 (22.3)	<0.001
	8	144 (96.5)	184 (91.0)	252 (84.7)	590 (77.7)	
Social activity score	<4	10 (5.4)	26 (12.3)	35 (10.4)	141 (17.6)	<0.001
	4	141 (94.6)	178 (87.7)	265 (89.6)	622 (82.4)	

Values are presented as unweighted frequencies (*n*) and weighted percentages (%). *p*-values were obtained using the Rao–Scott chi-square test. The LF-10 total score was converted from a 40-point scale to a 0–100 scale; a score of 100 corresponds to the maximum raw score of 40.

**Table 4:** Association between physical activity groups and life functioning.

	Inactive	Aerobic Only				Muscle-Strengthening Only				Combined			
	Reference	Crude	Model 1	Model 2	Model 3	Crude	Model 1	Model 2	Model 3	Crude	Model 1	Model 2	Model 3
No life functioning limitations (LF-10 total score = 100)	1	**1.68 (1.22–2.30)**	**1.60 (1.16–2.19)**	**1.55 (1.11–2.17)**	**1.55 (1.11–2.18)**	**1.94 (1.28–2.92)**	**1.77 (1.16–2.70)**	**1.69 (1.08–2.64)**	**1.65 (1.06–2.56)**	**3.74 (2.59–5.39)**	**2.45 (1.61–3.74)**	**2.20 (1.43–3.40)**	**2.19 (1.41–3.39)**
No lower extremity function limitations (LF-10 lower extremity score = 20)	1	**1.68 (1.23–2.28)**	**1.58 (1.16–2.15)**	**1.54 (1.12–2.13)**	**1.55 (1.12–2.15)**	**1.92 (1.29–2.85)**	**1.78 (1.18–2.68)**	**1.70 (1.11–2.62)**	**1.67 (1.09–2.56)**	**3.68 (2.05–5.31)**	**2.42 (1.59–3.70)**	**2.21 (1.42–3.42)**	**2.20 (1.42–3.42)**
No upper extremity function limitations (LF-10 upper extremity score = 8)	1	1.11 (0.83–1.48)	0.94 (0.70–1.27)	0.90 (0.67–1.22)	0.90 (0.67–1.22)	**1.83 (1.20–2.78)**	1.57 (0.97–2.55)	1.52 (0.94–2.47)	1.54 (0.96–2.49)	**4.11 (2.55–6.62)**	**2.19 (1.34–3.60)**	**1.88 (1.15–3.06)**	**1.87 (1.14–3.06)**
No IADL limitations (LF-10 IADL score = 8)	1	**1.60 (1.13–2.25)**	1.36 (0.95–1.95)	1.33 (0.93–1.91)	1.31 (0.91–1.88)	**2.91 (1.68–5.04)**	**2.72 (1.53–4.83)**	**2.49 (1.40–4.45)**	**2.36 (1.34–4.16)**	**7.96 (3.38–18.77)**	**4.69 (1.96–11.23)**	**3.69 (1.51–8.99)**	**3.71 (1.50–9.16)**
No social participation limitations (LF-10 social participation score = 4)	1	**1.85 (1.25–2.75)**	**1.58 (1.07–2.33)**	**1.53 (1.03–2.27)**	**1.53 (1.02–2.29)**	1.53 (0.90–2.60)	1.36 (0.76–2.44)	1.27 (0.72–2.25)	1.25 (0.71–2.22)	**3.74 (1.79–7.85)**	**2.21 (1.04–4.71)**	1.75 (0.80–3.86)	1.76 (0.79–3.94)

Values are presented as odds ratios (ORs) and 95% confidence intervals (CIs). Complex sample logistic regression analysis was performed. Crude: unadjusted; Model 1: adjusted for sex and age; Model 2: Model 1 + education level, household income, alcohol consumption, smoking status, and body mass index; Model 3: Model 2 + hypertension, diabetes mellitus, hypercholesterolemia, and hypertriglyceridemia. Bold values indicate statistical significance (*p* < 0.05).

**Table 5:** Sensitivity analysis of the associations between physical activity recommendation adherence and LF-10 scores.

	Inactive	Aerobic Only	Muscle-Strengthening Only	Combined
	Reference	*B* (95% CI)	*B* (95% CI)	*B* (95% CI)
LF-10 total score (0–100)	0	**3.31 (1.48–5.14)**	**5.55 (3.20–7.90)**	**4.26 (2.31–6.21)**
Lower extremity function score (0–20)	0	**0.88 (0.38–1.38)**	**1.42 (0.80–2.04)**	**1.20 (0.68–1.72)**
Upper extremity function score (0–8)	0	0.10 (−0.06–0.26)	**0.35 (0.14–0.55)**	**0.23 (0.07–0.39)**
IADL score (0–8)	0	**0.22 (0.09–0.35)**	**0.33 (0.18–0.47)**	**0.19 (0.04–0.34)**
Social participation score (0–4)	0	**0.13 (0.04–0.22)**	**0.12 (0.02–0.23)**	0.09 (−0.02–0.19)

Values are presented as unstandardized regression coefficients (*B*) and 95% confidence intervals (CIs). Complex sample general linear models were used. The models were adjusted for sex, age, education level, household income, alcohol consumption, smoking status, body mass index, hypertension, diabetes mellitus, hypercholesterolemia, and hypertriglyceridemia. Bold values indicate statistical significance (*p* < 0.05).

## Data Availability

The data analyzed in this study are available from the Korea National Health and Nutrition Examination Survey (KNHANES), conducted by the Korea Disease Control and Prevention Agency (KDCA). The data are available through the KNHANES website upon request and approval.
